# Supplementary material for: Genome-wide deposition of 6-methyladenine in human DNA reduces the viability of HEK293 cells and directly influences gene expression
Source: Commun Biol. 2023 Feb 2;6:138. doi: 10.1038/s42003-023-04466-1 (PMC9895073; doi:10.1038/s42003-023-04466-1)
Supplement: Supplementary file 2 — Supplementary Information [file 42003_2023_4466_MOESM2_ESM.pdf]

Genome-wide deposition of 6-methyladenine in human DNA reduces the viability of HEK293 cells and directly influences gene expression

Julian Broche, Anja R. Köhler, Fiona Kühnel, Bernd Osteresch, Thyagarajan T. Chandrasekaran, Sabrina Adam, Jens Brockmeyer, Albert Jeltsch*

Supplementary Information

Supplementary Figures

Supplementary Figure 1: m6dA-dependent genomic DNA (gDNA) digestion after expression of bacterial N6-MTases in HEK293 cells.

Supplementary Figure 2: Increased global m6dA deposition in HEK293 cell lines expressing two bacterial N6-MTases.

Supplementary Figure 3: Additional data related to the analysis of m6dA in genomic DNA by mass spectrometry.

Supplementary Figure 4: Identification of m6dA-responsive promoters in GFP reporter assays.

Supplementary Figure 5: Sequences of the cloned promoters responsive to m6dA deposition by CcrM.

Supplementary Figure 6: Data related to the H3K27me3 analysis at genes upregulated by m6dA.

Supplementary Figure 7: Reduction of JUN DNA binding by m6dA.

Supplementary Figure 8: Validation of genomic m6dA DNA methylation at promoters responsive to m6dA deposition at GANTC motifs.

Supplementary Figure 9: Flow cytometry gating strategies for the different settings of the competitive proliferation assays.

Supplementary Figure 10: Flow cytometry gating strategy utilized for the GFP reporter assay.

Supplementary Tables

Supplementary Table 1: List of differentially expressed genes and their expression in each condition.

Supplementary Table 2: Oligodeoxynucleotides used in this study.

Supplementary Figures

a

Restriction enzyme	Sequence	Enzyme activity
*Hinf*I	5‘-G^{m}ANTC-3‘	Blocked
	5‘-GANTC-3‘	Cleaved
*Dpn*I	5‘-G^{m}ATC-3‘	Cleaved
	5‘-GATC-3‘	Blocked
*Dpn*II	5‘-G^{m}ATC-3‘	Blocked
	5‘-GATC-3‘	Cleaved

b

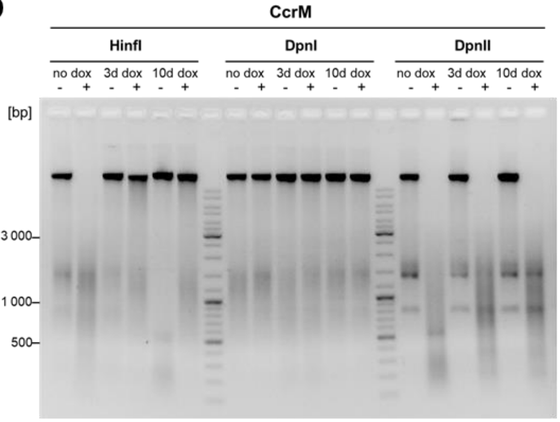

c

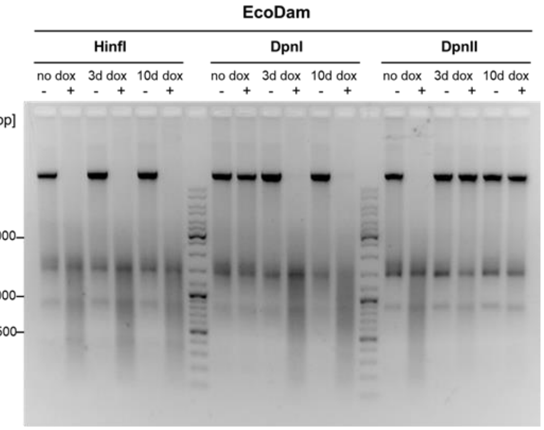

d

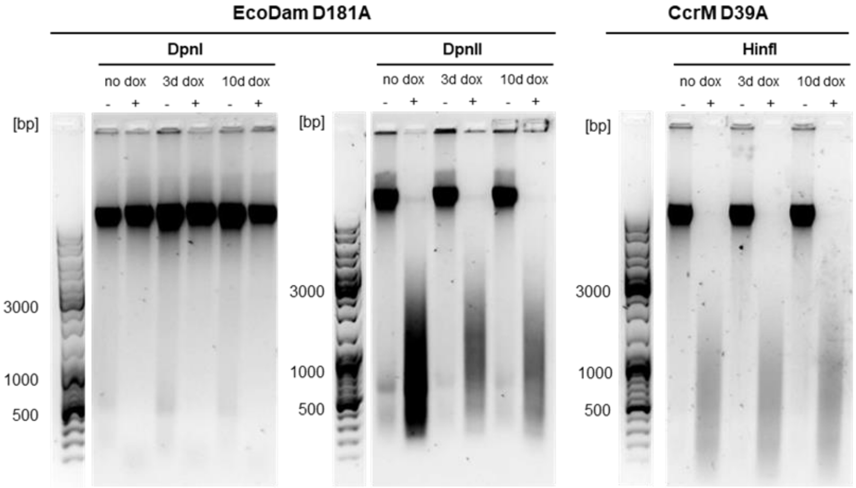

Supplementary Figure 1: m6dA-dependent genomic DNA (gDNA) digestion after expression of bacterial N6-MTases in HEK293 cells. a Table of restriction enzymes that were utilized for the digestion of the gDNA, their recognized sequences, and sensitivity towards adenine methylation. **b** Example of a 1% agarose gel showing the digested ('+') or undigested ('-') gDNA isolated from the stable HEK293 cell line containing CcrM. CcrM expression was either not induced ('no dox') or induced by doxycycline (dox) treatment for up to 10 days ('3d dox', '10d dox'). The digestion patterns indicate occurrence of global, genome-wide adenine methylation in GANTC motifs and absence of methylation in GATC motifs. **c** Same as in panel (**b**), but HEK293 cells with EcoDam expression were studied. The digestion patterns indicate occurrence of global, genome-wide adenine methylation in GATC motifs and absence of methylation in GANTC motifs. **d** Same as in panels (**b** and **c**), but HEK293 cells with inactive MTases were studied. CcrM D39A and EcoDam D181A are catalytically inactive MTase variants that carry an amino acid exchange in the active center. The digestion patterns indicate absence of methylation in GANTC and GATC motifs. Gels were stained with GelRed. Uncropped images of panel (**d**) are provided in Supplementary Data 1.

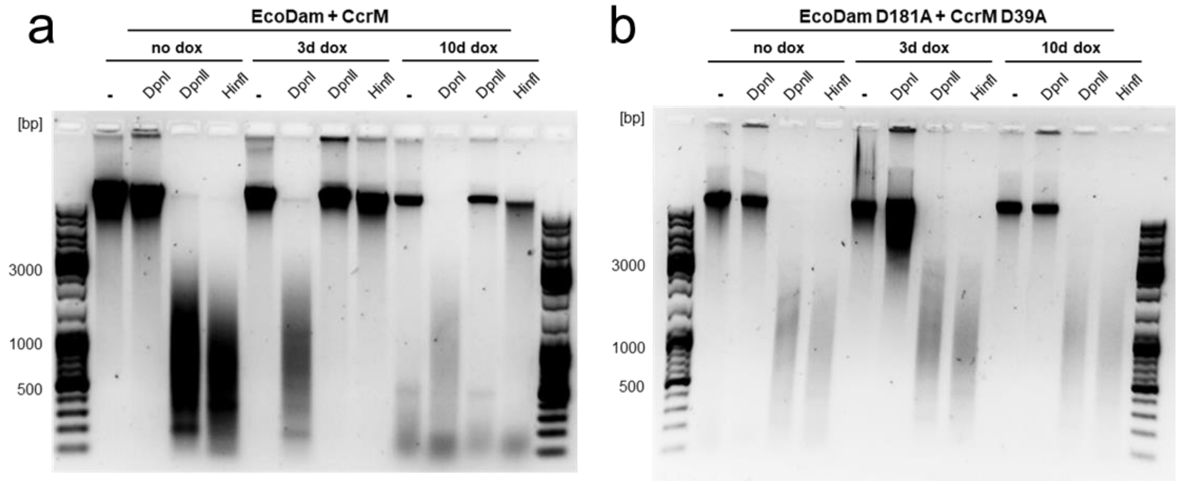

Supplementary Figure 2: Increased global m6dA deposition in HEK293 cell lines expressing two bacterial N6-MTases. a Example of a 1% agarose gel showing the digested (‘DpnI’, ‘DpnII’, ‘HinfI’) or undigested (‘-‘) gDNA of stable HEK293 cell lines after the co-expression of CcrM and EcoDam was induced by doxycycline (dox) treatment for up to 10 days. The digestion patterns indicate occurrence of global, genome-wide adenine methylation in GANTC and GATC motifs. **b** Same as in panel (**a**), but gDNA was isolated from cells that co-express catalytically inactive CcrM D39A and EcoDam D181A mutants. The digestion patterns indicate absence of adenine methylation in GANTC and GATC motifs. Gels were stained with GelRed.

a

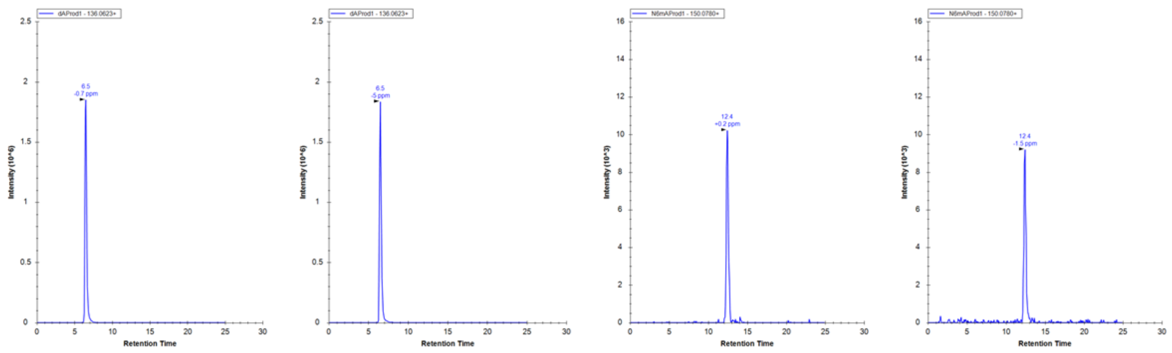

b

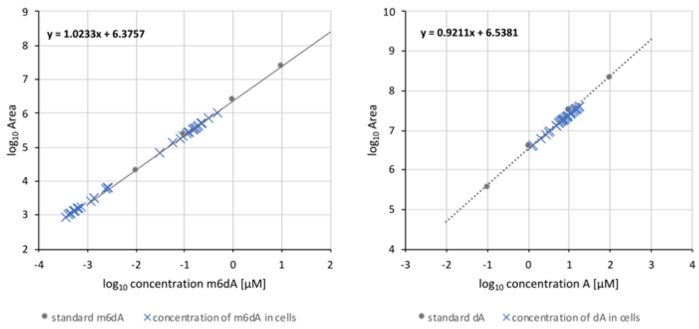

c

Nucleoside	Precursor ion [m/z]	Product ion [m/z]
dA	252.1097	136.0623
dC	228.0984	112.0511
dG	268.1056	152.0572
dT	243.0981	127.0508
m6dA	266.1253	150.0780

Supplementary Figure 3: Additional data related to the analysis of m6dA in genomic DNA by mass spectrometry. a Examples of LC profiles of standard peaks and peaks obtained with gDNA isolated from cells after 3 days induction of wildtype CcrM. **b** Examples of calibration curves for m6dA and dA obtained after HPLC-MS/MS measurement with the external m6dA and dA standards. The logarithmic concentration of the standards is shown against the logarithmic area under the curve as grey dots. The data points of biological samples are indicated as blue crosses. **c** Characteristic ion transitions resulting from the cleavage of the glycosidic bond in nucleosides that were used in this work.

a

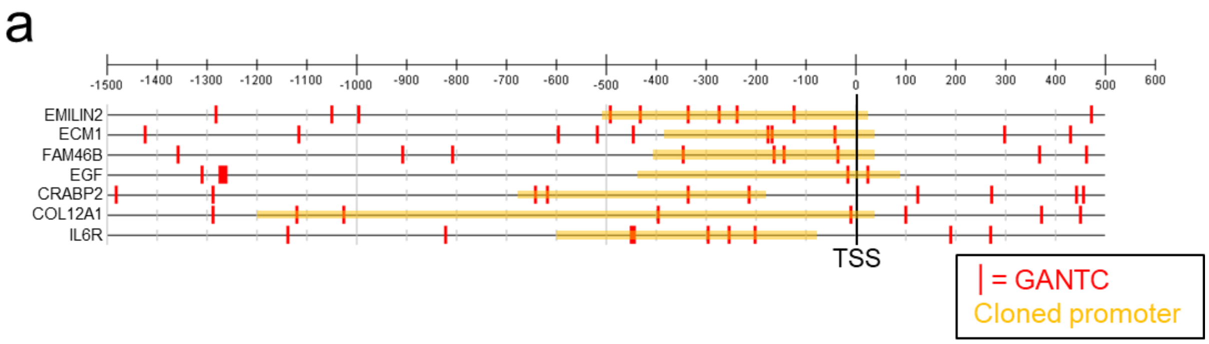

b

Cloned promoter	Coordinates (Hg38)	n (GANTC)	Normalized counts (DeSeq2) no dox CcrM rep. 1	no dox CcrM rep. 2	10d dox CcrM rep. 1	10d dox CcrM rep. 2	10d dox CcrM D39A rep. 1	10d dox CcrM D39A rep. 2	log2-fold change to 'no dox' 10d dox CcrM	10d dox CcrM D39A	P-value
EMILIN2	chr18:2846520-2847026	6	1754.0	1328.9	82.7	61.6	1243.0	976.3	-4.42	-0.47	5.3E-05
ECM1	chr1:150507733-150508135	3	92.2	158.0	9.4	10.3	170.1	153.1	-3.67	0.37	1.9E-02
FAM46B	chr1:27012820-27013260	4	263.7	296.4	37.7	33.9	254.5	207.0	-2.97	-0.28	1.1E-02
EGF	chr4:109912451-109912962	2	130.1	102.9	6.3	4.1	76.5	85.7	-4.49	-0.52	5.1E-03
CRABP2	chr1:156705763-156706261	4	783.5	514.3	166.4	163.1	847.0	623.9	-1.98	0.18	1.9E-02
COL12A1	chr6:75206037-75207226	4	456.8	640.6	1884.3	1530.7	382.4	683.6	1.64	-0.04	5.7E-02
IL6R	chr1:154404587-154405100	5	162.0	332.9	34.5	21.5	222.6	242.6	-3.14	-0.09	2.4E-02

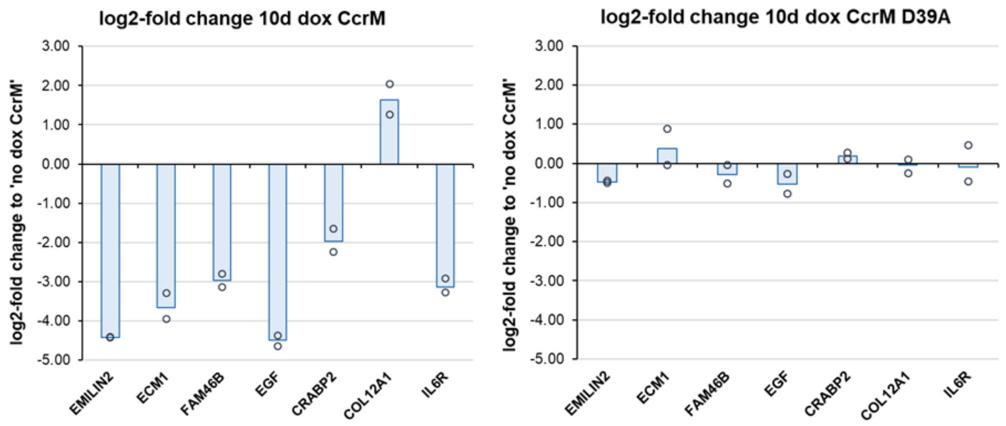

Supplementary Figure 4: Identification of m6dA-responsive promoters in GFP reporter assays. a Overview of selected gene promoters containing multiple GANTC motifs (red dashes), and their relative position to the transcriptional start site (TSS). The cloned parts of the promoters are highlighted in orange. **b** Expression levels of selected genes obtained by RNA-seq. Read counts were normalized by DeSeq2. The frequency of GANTC sequences and the genomic location of the cloned promoters are indicated in the table. Error bars in the bar diagram represent the propagated error of the standard deviations of two biological replicates.

EMILIN2 promoter:

CGTTCGTTTCCTCCTGGACTCTCTAGGTCCATGGAGCTGGTGGGGGAACGGCCCTGTCTTGCCCGAGGAGGAAATTCGAGTCGAGCCCCCACTCCTCTCCCCGCGGGACTGATAAACATGTCCCTCATTCGCC
CCGGGAGGCTGGCACCCAGGCACGACGCGAGGACGGCCAGACTCGCCGACGGCGGCCGCGTCCCGGGGGGCCGGGAGCGAGCCTGTCGGAAGGTGGGAGGCGGAGTCGCTCGGCATCTGGGAAGAGAC
CAAAGGAATGAGTCATTGGAAATGCTGTAACGTAGACCCTTATCCCAAACCCTTTCGCCCCTCCACCCCGAGCCCCCTGCCGAGAAGCGAAGGGACTGGAGACGGGGAGACTTGGTGGGGGGAGAGTCACGTT
TCGGAGAGGGAAGTGAAATAAAGAGGGCGGCACCCGGGGGGACCTGTGCGTCATTGAGGGACCGGGGAGAAGCGCAGGGCGCACGGGGCTGC

ECM1 promoter:

GGGGAGATCCCTTGGATAGGTTTCCTGCTCCCCGCTGTAAACTCCTTTGTGCCCTGTTTCCCCAACCCCAGTTCATGAGTTGGCAGATCCCCTCTCCACAGAAGGCAGCCTGTAGATGGAGAAACTGAGGCACA
AACTAGGGGCTCAAACACCTCTTGCTCCTGGATTCTCTGAATCAGTTTCTCTTGAAGGTCATAAGAATGTTCTCCTCTAATCAGTCCCATTCTTCTCAATCCTCTGAGGAGGCTTCAGGGTGACTTTGGGAGGAGT
GGCTTCCAGAGGAGGAGCAGCTGGGACTGAGTCATGGCAGGAAGCTGAGGAGGGCGGGAGATCACACCAGACAATTATAAAAGAAGAGCTGGTCCTGAAGCTCACAACCGTAACAGCCACCAGACAAGCTTC

FAM46B promoter:

GACAAGGCTAGGTGACTTCTCAATTTCTTCCAGCGCTAAAAAAAAAAAACAGAAACCTGGAATTTCTCAGTGGTCTGACTCTGCTACAAACTCCGCAGGTCACTTCCCTCCCTCCTGACCCTGGCGGGGCGGGGT
GGGCGCTGTCCCTTTAAGTCCAGGCCCGGGCCAATCACAGTGAACAGAAGTTCTTTAAGGACCAAAGGAGGGAATTCAGCCTGAGAAGGAGGGCGGGGCAGGGGCGTGCCCTCTAGCCAATCGGAATCTCCC
TAGAGAACAGTGACTCACGTGCAAAGCTCTACAGACCAGTGTCCGGAGCGTTCAGAGGCGGGGCGGCTCTCGCTGTTGGCAGCGGCCCGGACCAATGGGCACGTCGCCCGCGGCCTATAAGAGTCCGCTGG
GCCGCGGGCTGCAGGCGCTAGGGTCGGTTAGTCGTCGCCGTCT

EGF promoter:

CTCCTGGAATGTGCACTGGTATTGACATTTCGTTCAAATAATGGGCTGAAGGTGAACTATCTTTACTATTGCTCATGTGAATGATCAATTATTATCAATCCAAACTATCAACAGTGCTGCTTATTATTGATTTATACTG
CATGGTTTAATTTTGTCTCCATCCTTCCAGACATTAAAACATAGCCAATATTTAGCAGTTCCCGCCATTCACCATGAGCACCTCCACAGCCCCCAACTCCTCTCTCCCAGTTCTGCCCAGGATATTTATGGTGAGTA
GCGAGTTATCTCCTCTTTGGCAGTCATCCCTGCTTTCCTGTGTGGAGGAATTGCCCACATTCGCATTTGCAAACAGAGGCTCACTCAAGTGTCACTAAAGGAAAGGAGGTGGAGCCTGAAGAGCTTTAAAAAGCA
AAGCTGAGTCATTCCACTTTTCAAAAAGAGAAACTGTTGGGAGAGGAATCGTATCTCCATATTTCTTCTTTCAGCCCCAATCCAAGGGTTGTAGCTGGAACAATTGA/TTTCCA

CRABP2 promoter:

GCGCATTAAGGGGAGCGAGTCGCCTGGCGACTACTTCCAGAGTCCCCAGGCATTACGTGAGCCCGAAGCAGGGTGGAGGGGTGGGGGGACCGTGCCGCCCCCGCCCAGCCTCTCCGAGTTGTTCCAGCAGG
GGGCGCCGTTGCCTCACTTAGATCCCTAACCCCCGGAACCCCGCAGCTCCCAAGCCCCTCTCTGAGTACGGAGTGGTCCCACTGGATCCAGTTCAGGGTTCAATGGAGCTAGGGCCAGCTACGGCTCAAGATC
TGGGGTCCGCCTGCGGGTGGGGTCGCCAGGTGTCCGGCACCAAGGAGTTGAATGCACCGAGTCAGGTTGGGGATGGGTGGGGAACAGGCGAGACGTGAGGAACTCGGGTGGGGGACAGCCATACACGAGC
CCTGAGCATCTGCGCCCGCAGCTAGCTCCCCCCGCCTCTGCGGAGAGCGCGATTCAAGTGCTGGCTTTGCGTCCGCTTCCCCATCCACTTACTAGCGCAGGAGA

COL12A1 promoter:

CTTCAAGAGGCCAAAACGGTGCAGGGCCTATACTGAGGTCAGACGTTATAATCAGAATCTCTCCAGTAGCATTTTTCTGATTTCTTGGTGTGAGCGCTCGAAGCTTCCTCCCGTGCGCCTTGTACAATCTGGCAAC
CCTGAGTACCAGACTCCCTGCCCAGGAACAAGGGGTAGGATGCCAGGATGGAGGGGGCTGTAAGAAGAGGCGACGTTGTTGGTAGAATATCAGCCCCCTATTTGGCTTCTCCAGTACTGTGTGTGGAGGGGTG
CTTTGGGGTTTCAAACTTACTCCTCTTCGCCCCTTTGGCTCCGCGAGGTTTTAAGGGCAGAGCTTTCAGTCCGAAGCCTCCGCTCGGGGCCAGGTTTTGCTCTGGTCCGGGTGAGAAGTTTCTGCAGTGGATGC
AACTTGTTACTGAGCGGCTTCCTATTCTGAGAACTCCTGGGTTTCATAGGTTTCCTCTCCCTCTCTTTTGGCTCAACTTAAGCCTCCCAGTTCCGTCCTGAAGCACCAGGGGAGTGAGAAAGGACTGGGAAAGCC
ATCCCATCACTGAGCCCCGCGCGGGCGGCTGTCCGGGTTTCCAATCGCGCTTGGAGATTGAAAAAGACAGAAGGCGCGCCGCGTGTACAGACAGTTAGTTAATGGAAATCCTGTAAATCGGTTTTAAGTGCACC
CAGGACAGAGGGGGAGGAAGATGGAGGTGGTGATATCAGATGAAGGATGGAGATCCGCAGGAGCGGGGGTGAATGGGTTACGCATCCTTGCATTTCCGAAAATGGGAGTCGGTCCCCTCCCCATTGTTCTTAA
TGTAGAAAAATTCATTAAAGAAATCTCAGGGATAGGGAAACCCGAGCTTCATTAAAGAAATCCCAGTCCGCCCCAAGTGGCGGAGTACGCGGCTTCCCTTGGCAGGGAACCCGGCTGCGGCTGTCCCCCTCTAC
GCTCCCTCCCTTCTCCCCGCCCCCCAGCGGGGCACTGCTACTACGGCGATCGCCACCTCACGCCGAAGGAATGCTCGAAGTATGCACACGTTCCCAAAAGTAGACCTCCTTCACCGCCGGAGGAGCATACATC
ACCCCGTGGCAGCCGTCCACCTTCTCAGGCGTTTCGTAACCGAGTAAACTGAGAGCTGGGAACAGCCCTCTATTAAGTAGCCCGCGGCGCGGAGTCTCTTTCCACTTGGCTAAGGCGA

IL6R promoter:

TCTGTCAAGTCAGGAGGGCAGCCATGAACGTTCTGATGTCTACTGAGCACGTGTGGCCCAGACCGTGTGTCAGGTGTTTAGGTGCCATCCACAGAACCTTCCTAATAACCCTGGGCAGCATAGGCTTTCTTATCT
CTGACAGATGAGGAAATGGAGACTCAGATTCTGAACCGAAGTCACAGACACAGTAGATGGTAGGTCTAAATGGGGACCCAGGTCTATCTGACTGCAAAGTCCAAACCGTTTCCTTGCCTCTGCTGCAGCCTGCG
AGGAGCAGCTGGGCAGAAAGACTGTGCCTTTACGGTGGTGAGTCTTCCGATGCCCAAGCCTCACCCCAGACCGATGAAATCAGAATCTCTGGAGACCCGACCCAGACATTGGTGGGTTTTAGGGCTCCTGGCT
GATTCCAGTGGGCAACCGGGCTACCAAGCACTGTTTTACCCTAAGGGTAAGAGGAACATCGCCTCCTCGAGGGCTCTCCAGGTGTGCCTTTCACAGCGTAATCCCGTTCACG*AAACCAAAAATCAAAACTAGGC*
CACTGCCCACTGGTGGCTGATACGCCCTTTTCTCATCAACAGAACCGGGAGGAAGGGCTCTGGTGGGGCTGGCGGTCCCCTGTTCTCCCCGCTCAGGTGCGGCGCTGTGGCAGGAAGCCACCCCCTCGGTCG
GCCGGTGCGCGGGGCTGTTGCGCCATCCGCTCCGGCTTTCGTAACCGCACCCTGGGACGGCCCAGAGACGCTCCAGCGCGAGTTCCTCAAATGTTTTCCTGCGTTGCCAGGACCGTCCGCCGCTCTGAGTCA
TGTGCGAGTGGGAAGTCGCACTGACACTGAGCCGGGCCAGAGGGAGAGGAGCCGAGCGCGGCGCGGGGCCGAGGGACTCGCAGTGTGTGTAGAGAGCC

Supplementary Figure 5: Sequences of the cloned promoters responsive to m6dA deposition by CcrM. GANTC motifs are shaded in yellow, TGANTCA motifs have an additional green shade. The A residues mutated in TGANTCA motifs in the EMILIN2 promoter are printed in pink. Underline indicates 5'UTR, parts printed in grey were not included in the experimental promoter construct. qPCR amplicons containing CcrM motifs are printed in red. The control amplicon without CcrM motifs is printed in blue.

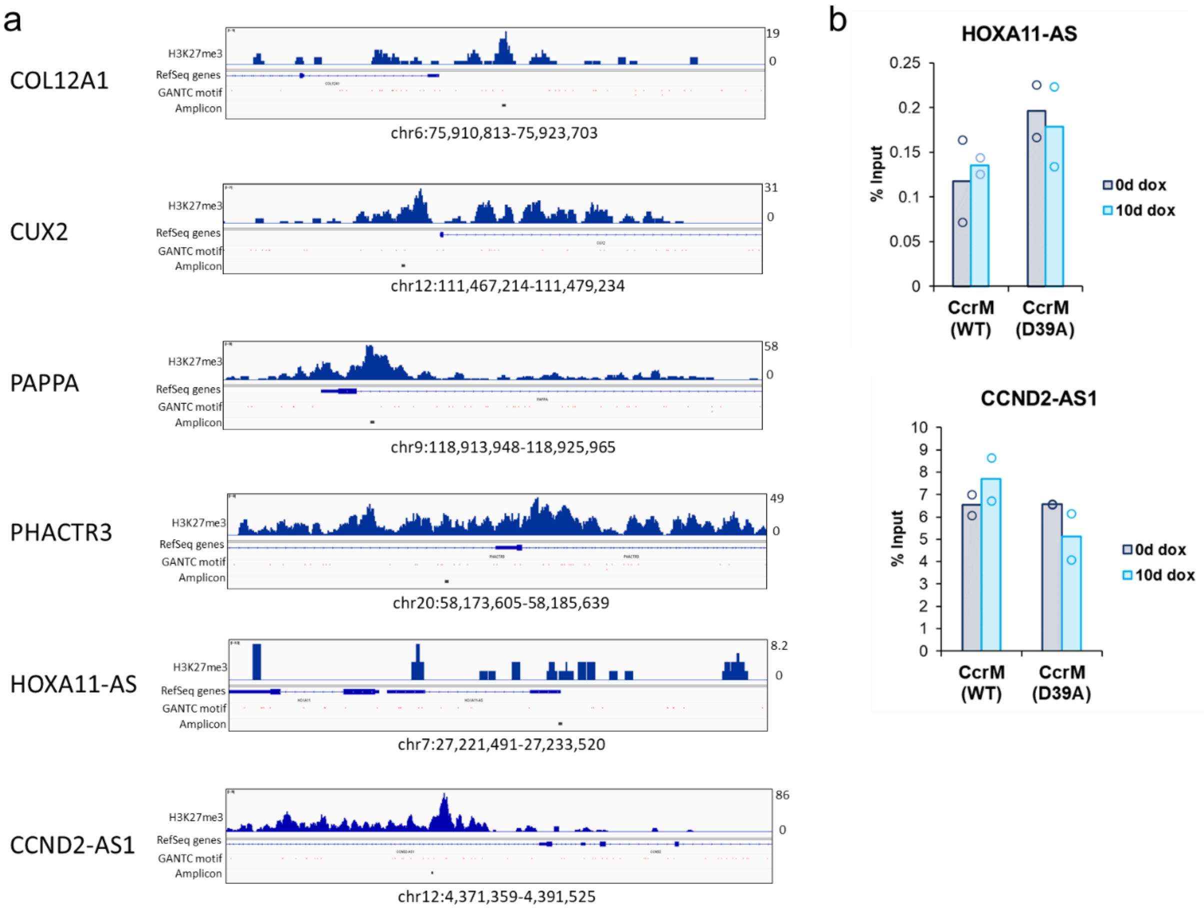

Supplementary Figure 6: Data related to the H3K27me3 analysis at genes upregulated by m6dA. a Genome browser views showing the genomic loci used for H3K27me3 ChIP-qPCR including H3K27me3 ChIP-seq data [1], locations of the genes, GANTC motifs and qPCR amplicons (from top to bottom). **b** Result of the H3K27me3 ChIP-qPCR experiment at the *HOXA11-AS* and *CCND2-AS1* loci showing absence of m6dA-dependent H3K27me3 changes. These regions were used as negative controls for the H3K27me3 ChIP-qPCR experiments, because expression of the corresponding genes did not change after m6dA deposition. The results of two technical replicates from independent qPCR runs are depicted as dots with the average shown as bars.

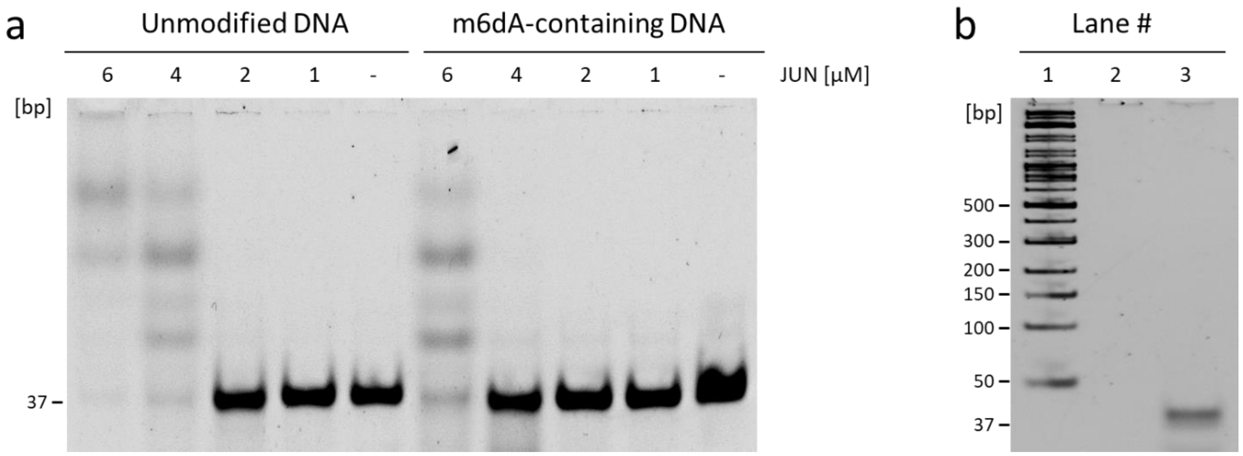

Supplementary Figure 7: Reduction of JUN DNA binding by m6dA. a Two 37mer DNA molecules containing one JUN binding site either in unmodified state or with m6dA modification (Supplementary Table 2) were incubated with different concentrations of purified JUN protein. DNA binding was analyzed by a band shift assay. The figure shows the fluorescence signal originating from the Cy5 label of the DNA captured by a Fusion advance solo 4 (PeqLab). **b** Gel run under identical conditions showing the free unmodified DNA from panel (**a**) (lane 3) together with a DNA size marker (lane 1). Lane 2 is empty.

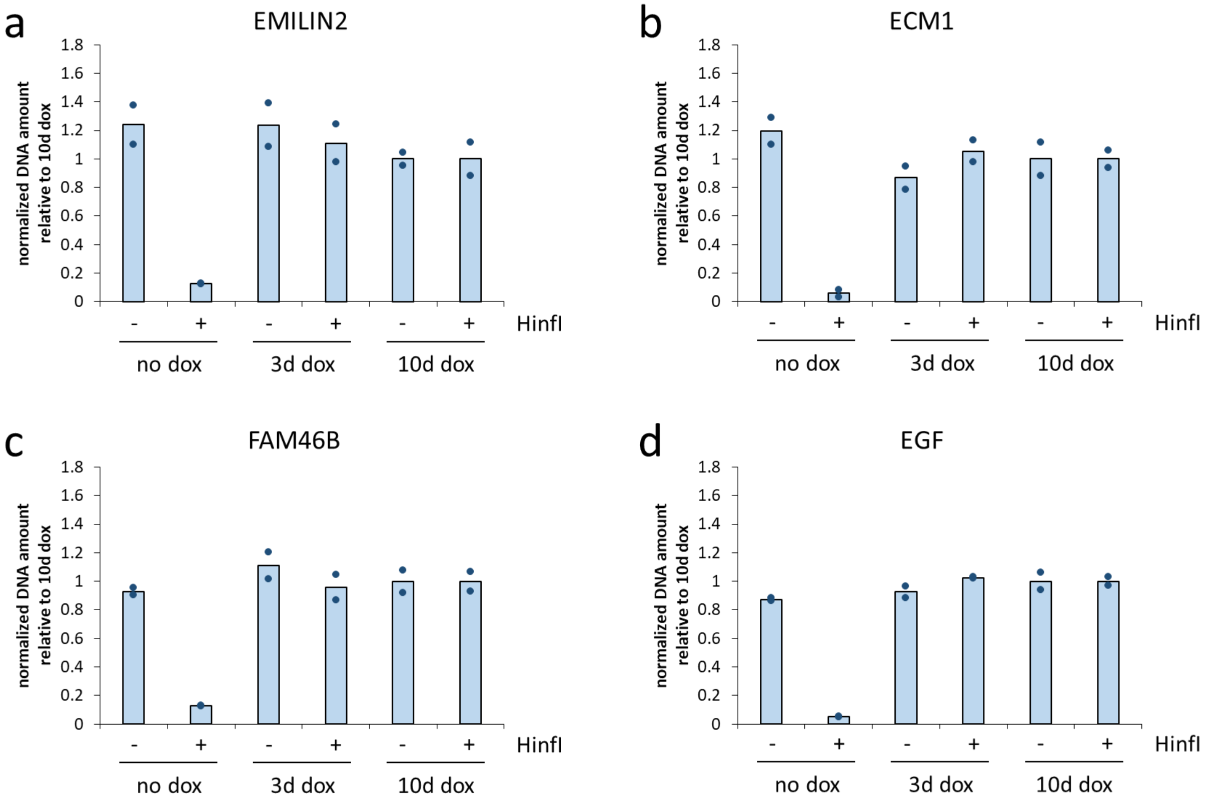

Supplementary Figure 8: Validation of genomic m6dA DNA methylation at promoters responsive to m6dA deposition at GANTC motifs. Genomic DNA isolated from no dox, 3d dox and 10d dox cells expressing CcrM were digested with HinfI. DNA amounts in digested and undigested DNA was investigated by qPCR using primers specific for amplicons from the EMILIN2 (a), ECM1 (b), FAM46B (c) and EGF (d) promoter regions that contain at least one HinfI site (see Suppl. Fig. 5). The results of two technical replicates from independent qPCR runs are depicted as dots with the average shown as bars.

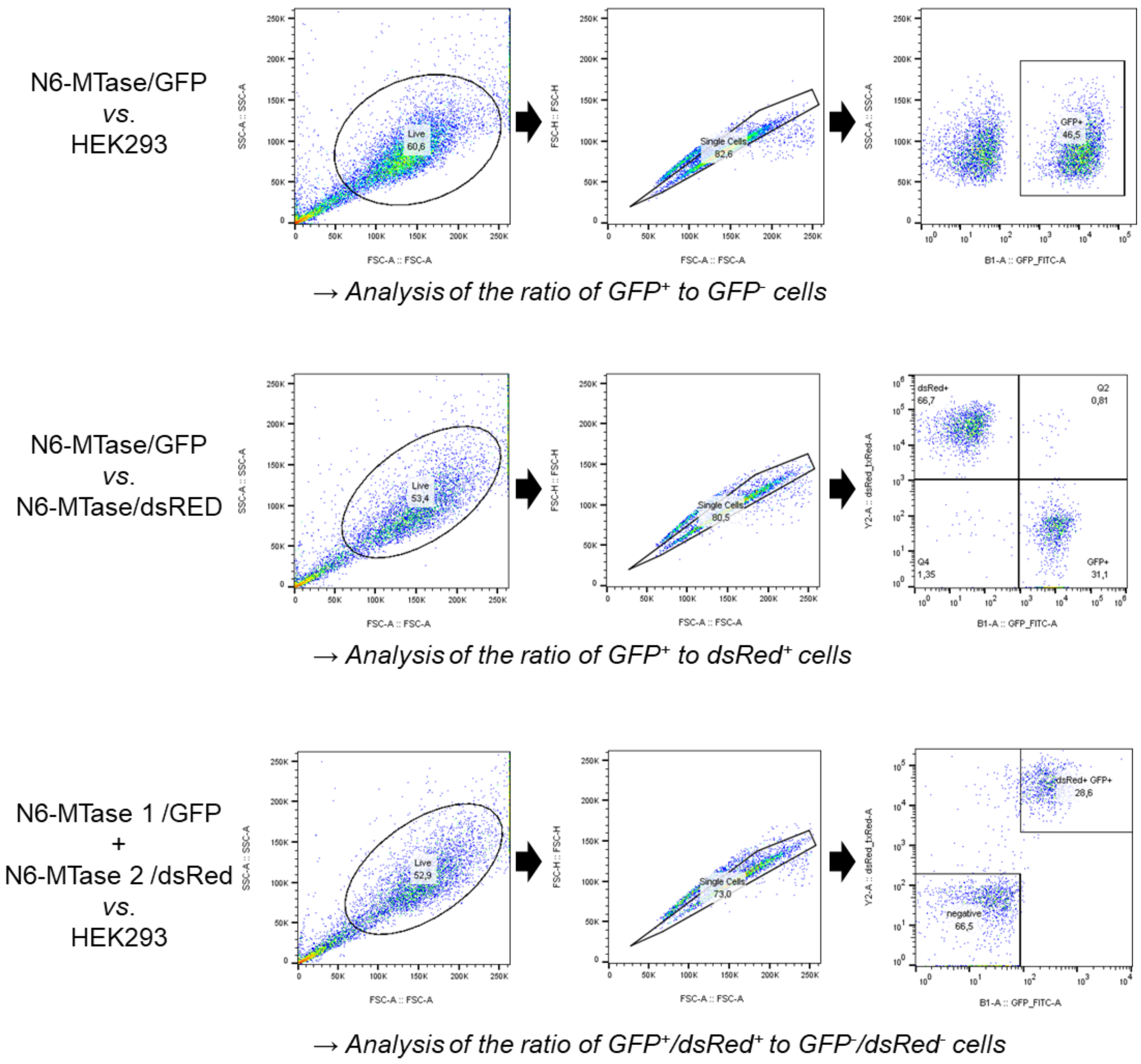

Supplementary Figure 9: Flow cytometry gating strategies for the different settings of the competitive proliferation assays. Depending on the system, stable HEK293 cell lines were used that contained constructs for expression of the N6-MTases with co-expression of GFP or dsRed via an IRES. FSC-A = forward scatter area, FSC-H = forward scatter height, SSC-A = side scatter area, GFP$^+$ = green fluorescent protein positive cells, dsRed$^+$ = red fluorescent protein positive cells.

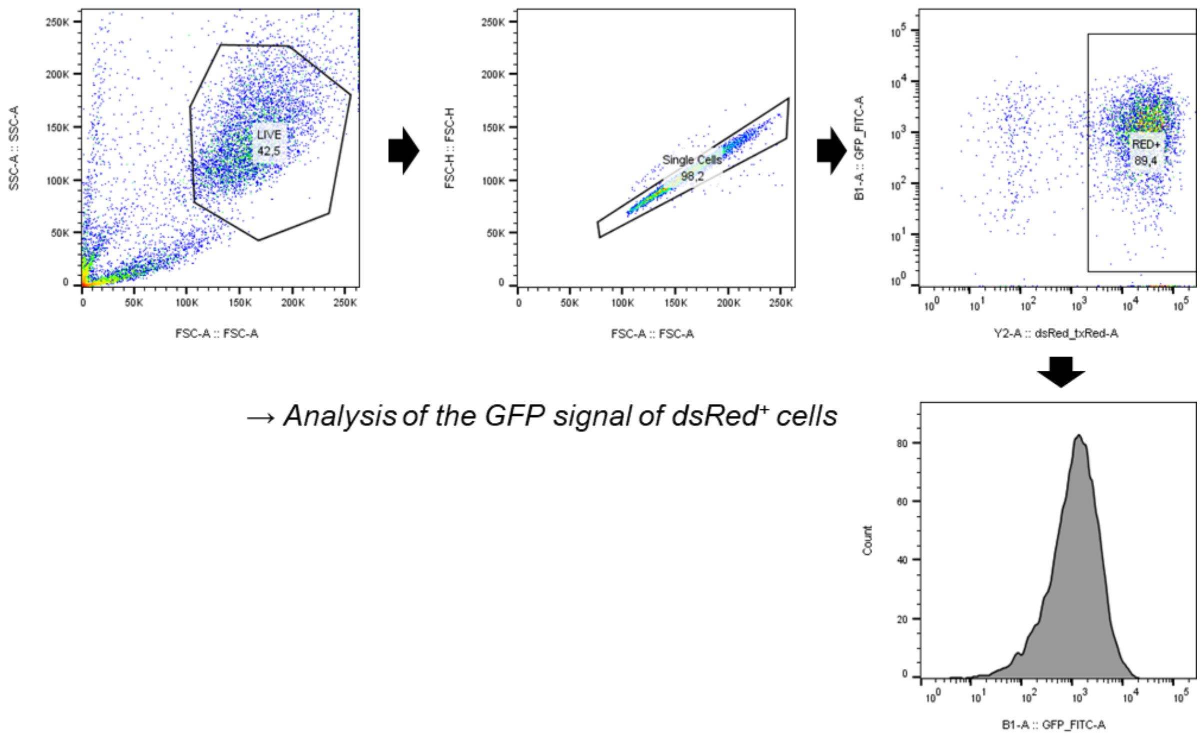

Supplementary Figure 10: Flow cytometry gating strategy utilized for the GFP reporter assay. Stable HEK293 cell lines contained expression constructs for CcrM wildtype or D39A mutant co-expressing dsRed via an IRES. Reporter gene activity was determined as the median GFP fluorescence signal in living single cells which were dsRed positive indicative of the presence of the CcrM construct. FSC-A = forward scatter area, FSC-H = forward scatter height, SSC-A = side scatter area.

Supplementary Tables

Supplementary Table 1: Lists of differentially expressed genes and their expression in each condition.

Downregulated genes	DeSeq2 normalized counts		
	no dox CcrM	10d dox CcrM	10d dox CcrM D39A
CATSPER1	26.2	1.1	32.7
ECM1	125.2	9.9	161.7
EMILIN2	1541.5	72.2	1109.8
EGF	116.6	5.3	81.2
LINC01411	37.5	3.2	43.8
BAIAP2L2	41.1	3.7	42.6
IL6R	247.6	28.1	232.7
CPA4	87.2	17.2	138.8
TRPV3	167.7	22.9	156.2
TENT5B (FAM46B)	280.2	35.9	230.9
SYNDIG1	149.3	14.1	82.0
AXL	252.4	54.0	304.8
SYTL1	126.2	16.7	89.4
EEF1A2	1112.5	176.3	874.3
EMP1	218.0	34.9	165.2
CRABP2	649.0	164.8	735.5
BIN1	201.3	47.2	208.9
CYP4V2	275.0	76.7	300.1
PCYT1B	684.2	168.2	648.2
EMP3	329.8	118.2	445.6
SLC22A17	79.7	20.8	76.7
SYTL5	207.7	39.5	140.3
CAVIN1	5639.9	2136.3	7487.8
B4GALNT1	506.6	160.4	524.4
S1PR3	118.6	37.9	123.0
ZNF793	149.8	43.6	139.7
OLFM2	224.1	107.4	328.7
CAVIN3	311.2	103.2	297.9
UPP1	441.8	180.2	493.0
PLPPR2	577.8	225.5	601.5
PAX7	210.3	73.6	193.5
CSDC2	224.2	109.3	284.8
GPAT3	236.8	95.3	245.7
COL5A1	4391.1	1126.1	2897.2
LAMB3	319.0	98.6	251.3
MYRF	337.7	132.2	335.9
PDZD4	340.6	124.1	313.5
ENO2	558.0	224.9	566.0
PLPP7	136.7	50.3	125.6
GORASP1	460.6	203.7	475.4
ANK1	152.6	56.5	131.6
S100A16	572.0	307.8	714.7
CXXC5	740.4	309.4	699.3
TGFB1	1989.6	1134.3	2533.2

NES	543.9	196.6	437.8
MSX2	718.0	311.9	692.6
S100A10	2985.6	1603.8	3543.8
CLU	4746.6	1910.0	4198.9
ZNF395	1008.1	441.6	936.6
PRXL2A	706.7	340.2	714.1
NME4	3633.6	2171.2	4426.3
GNE	555.6	216.6	435.1
DFFA	3025.2	1743.6	3436.0
EFEMP2	536.0	250.3	490.3
BHLHE40	483.7	246.5	479.1
NQO1	342.3	156.5	301.2
DPF1	313.0	162.3	305.3
HEXB	1183.2	579.1	1084.8
PERP	2771.6	1635.3	2945.5
GSTP1	14188.6	9264.5	16590.1
TTC39A	598.2	350.2	614.1
FZD2	1008.7	563.5	980.2
BASP1	4027.1	2626.0	4566.1
ANXA2	7223.7	4589.1	7676.4
PHTF1	791.7	409.3	678.3
DARS2	2279.4	1318.0	2139.7

Upregulated genes	DeSeq2 normalized counts		
	no dox CcrM	10d dox CcrM	10d dox CcrM D39A
TARBP2	857.6	1362.5	830.3
CHEK2	505.1	826.1	477.5
PYCR1	1553.6	3122.7	1774.5
SPINT1	431.0	758.6	427.5
KIF1A	323.0	581.9	320.1
ABCB8	529.3	992.2	516.9
MAL2	424.2	838.7	436.4
NAIF1	189.6	345.2	175.4
FSTL1	3732.2	7118.5	3558.3
UBE2QL1	584.7	1304.8	649.1
BMP2	763.4	1521.4	728.3
ZSCAN20	193.1	391.4	186.1
MGLL	132.5	389.1	180.2
TIMP3	495.0	972.2	447.3
NXT2	253.8	523.6	236.6
ACSS1	258.5	503.7	220.9
SPTB	406.6	709.2	307.5
BOLA1	167.3	448.1	192.3
ALDH1A2	119.5	271.0	116.1
DUSP5	115.9	365.9	151.4
DENND2A	53.3	171.5	70.0
CDH13	131.5	287.2	110.9
SLC1A1	169.5	369.5	138.0
SHC2	95.5	204.1	75.6
FLT1	111.1	394.2	144.0
FBXL19-AS1	95.6	321.8	103.5
COL12A1	548.8	1707.6	533.1
ADGRL3	24.0	85.5	24.6
IL2RB	16.6	87.6	20.6
PAPPA	30.8	111.9	25.2
TRPC4	8.6	53.0	11.6
CUX2	43.5	180.8	21.7
PHACTR3	6.3	72.2	5.1

Supplementary Table 2: Oligodeoxynucleotides used in this study.

**Primers for H3K27me3 ChIP-qPCR**	
COL12A1_FP	CAAATAGGAATCGCCGCCTC
COL12A1_RP	TGTGTAGGCTCTTTGTGCGAG
CUX2_FP	CCCCCTCCCCGCCAATAATA
CUX2_RP	CCACTGAGTCGTGGGAAATGT
PAPPA_FP	TGCTCCGAATGGTCAGATGC
PAPPA_RP	ATCAACGGTCCCCAAGTCTC
PHACTR3_FP	CCCAAAGCTCTGTGTGGAGT
PHACTR3_RP	GGTTAAGCGCCAAGGACAGA
HOXA11-AS_FP	TTTCCACAGCCTTTGCAGGCG
HOXA11-AS_RP	TTCAGGCTGCAAGAAGAAGCGXG
CCND2-AS1_FP	CGCCTTCTTAACTCACGCCT
CCND2-AS1_RP	CTTCGGCGAATTTCGGCTTG
**Primers for m6dA sensitive qPCR**	
Emilin2_FP	GAGCGAGCCTGTCGGAAG
Emilin2_RP	CGAAAGGGTTTGGGATAAGGGT
ECM1_FP	AGGGGCTCAAACACCTCTTG
ECM1_RP	TTGTCTGGTGTGATCTCCCG
FAM46B_FP	GGGCGTGCCCTCTAGC
FAM46B_RP	GCGACGACTAACCGACCCTA
EGF_FP	TCCCAGTTCTGCCCAGGATA
EGF_RP	ACGATTCCTCTCCCAACAGT
Control_FP	ATCCCAAACCCTTTCGCCC
Control_RP	CAAGTCTCCCCGTCTCCAGT
**Oligodeoxynucleotides used for DNA binding analysis**	
Upper strand	Cy5-AAGAGACCAAAGGAATGAGTCATTGGAAATGCTGAGA
Upper strand Me	Cy5-AAGAGACCAAAGGAATG^{6m}AGTCATTGGAAATGCTGAGA
Lower strand	TCTCAGCATTTCCAATGACTCATTCCTTTGGTCTCTT
Lower strand Me	TCTCAGCATTTCCAATG^{6m}ACTCATTCCTTTGGTCTCTT

Supplementary references

1 Broche, J., Kungulovski, G., Bashtrykov, P., Rathert, P. & Jeltsch, A. Genome-wide investigation of the dynamic changes of epigenome modifications after global DNA methylation editing. *Nucleic acids research* **49**, 158-176 (2021).
