# Supplementary material for: Genome-wide deposition of 6-methyladenine in human DNA reduces the viability of HEK293 cells and directly influences gene expression
Source: Commun Biol. 2023 Feb 2;6:138. doi: 10.1038/s42003-023-04466-1 (PMC9895073; doi:10.1038/s42003-023-04466-1)
Supplement: Supplementary file 3 — Description of Additional Supplementary Data [file 42003_2023_4466_MOESM3_ESM.docx]

**Description of Additional Supplementary Files**

**File name:** Supplementary Data 1

**Description:** The source data behind all graphs in the paper
